# Intelligent Orthopedics: Machine Learning in Diagnosis of Bone Disease, Implants, and Bone Health Monitoring

**DOI:** 10.1002/adhm.71447

**Published:** 2026-07-19

**Authors:** Maryam Kamaei, Hesam Mohammadi, Shamim Golafshan, Mojtaba Alehosseini, Mahsa Mohammadzadeh, Atefeh Golshirazi, Nasim Golafshan, Tatiane Eufrásio‐da‐Silva, Alireza Dolatshahi‐Pirouz, Gorka Orive

**Affiliations:** ^1^ Nanotechnology and Advanced Materials Department Materials and Energy Research Center (MERC) Karaj Iran; ^2^ Department of Materials Engineering Isfahan University of Technology Isfahan Iran; ^3^ Knight Foundation School of Computing and Information Sciences Florida International University Miami Florida USA; ^4^ Department of Informatics, Bioengineering, Robotics and Systems Engineering (DIBRIS) University of Genoa Genova Italy; ^5^ Department of Health Technology Technical University of Denmark Lyngby Denmark; ^6^ Basque Sustainable Pharmacy & Biotherapy Research Group School of Pharmacy University of the Basque Country (UPV/EHU) Vitoria‐Gasteiz Spain; ^7^ Bioaraba Health Research Institute Vitoria‐Gasteiz Spain; ^8^ Regenerative Medicine BTI Biotechnology Institute Vitoria‐Gasteiz Spain

**Keywords:** bone health monitoring, intelligent diagnosis, intelligent implants, machine learning, orthopedics

## Abstract

Bone regeneration in orthopedics is a great challenge, as efficient recovery affects patient's quality of life. Common methods encounter different limitations regarding diagnosis, implantation, and bone health monitoring. Recent advances in artificial intelligence (AI), specifically machine learning (ML) algorithms, have presented opportunities to enhance these aspects by accurately analyzing imaging data. This provides detailed evaluations that help to determine bone condition and guide treatment strategies. ML models can facilitate a patient‐specific approach to select and design intelligent implants by examining extensive biomaterial datasets to identify the best match for each patient's biomechanical and biological needs. Furthermore, ML models can analyze data to accurately predict bone‐healing timelines, allowing clinicians to track recovery trends, and make timely interventions in potential complications. In clinical settings, ML tools assist with preoperative planning, postoperative follow‐up, and the design of intelligent implants, offering more effective, data‐driven decision‐making and favorable outcomes in bone regeneration. Looking ahead, while ML advancements show promise for effective orthopedic therapies, challenges persist. ML could revolutionize bone regeneration in various ways and offer predictive insights that promote patient‐centered orthopedic care. Altogether, considering the continued evolution of ML, its integration into clinical applications will be crucial for developing truly intelligent bone regeneration therapies.

## Graphical abstract

Efficient recovery from traumatic or degenerative diseases is a great challenge, even after all the advancements in bone and cartilage regeneration. Machine learning (ML) algorithms have presented opportunities to enhance these aspects by accurately analyzing imaging data. In clinical settings, ML tools assist in preoperative planning, postoperative follow‐up, and the design of intelligent implants, offering data‐driven decision‐making and favorable outcomes.

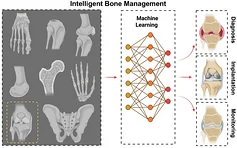

## 1 Introduction

The rising incidence of bone injuries and diseases increases the need for effective bone regeneration strategies. Bone loss due to degenerative disease and aging requires timely clinical intervention [1, 2]. Therefore, orthopedic approaches, such as autograft and synthetic implants, have recently been utilized [3]. These approaches, however, face some limitations, like the lack of diagnosis in the early stages of the disease [4], incomplete bone healing [5], and the absence of personalized therapies [6], encouraging researchers to seek new solutions. For example, dual‐energy x‐ray absorptiometry (DXA) and computed tomography (CT) scans are commonly used for osteoporosis diagnosis, as they provide detailed images of bone structure and fractures [7]. Despite recent studies and clinical trials in orthopedics, it is still challenging to diagnose bone disorders, optimize advanced designs and biomaterials, and monitor bone healing and its complications.

Diagnosing bone disorders remains a challenge, particularly due to limitations in conventional methods, especially low‐quality imaging techniques [8]. For instance, prenatal diagnosis of bone dysplasia relies heavily on imaging techniques like magnetic resonance imaging (MRI) and computed tomography (CT) scan [9]. With the current limitations in detection methods for early symptoms, improved diagnostic criteria are needed [10]. Another challenge is selecting appropriate materials and designs for bone implants, which requires mechanically stable, biocompatible materials [11]. Another challenge is bone healing, which involves mechanical and complex biological processes that can be approached using various methodologies [12]. The methods for monitoring bone healing are often limited in precision and reliability because they depend on clinicians’ interpretation of the bone‐healing process, causing the interpretations to be inconsistent [13].

These limitations highlight the need for intelligent bone‐regeneration methods. One revolutionary method is the application of machine learning (ML), a modern technique that offers substantial advantages over traditional methods for diagnosing bone disease, optimizing implants, and monitoring bone healing. Figure 1A shows how ML can process patient data to help clinicians manage bone diseases. Given its development and potential for accurate data processing, researchers have increasingly studied ML applications in orthopedics, results of which are presented in Figure 1B [14, 15, 16, 17, 18, 19, 20, 21, 22, 23, 24, 25, 26, 27, 28, 29].

**FIGURE 1:**
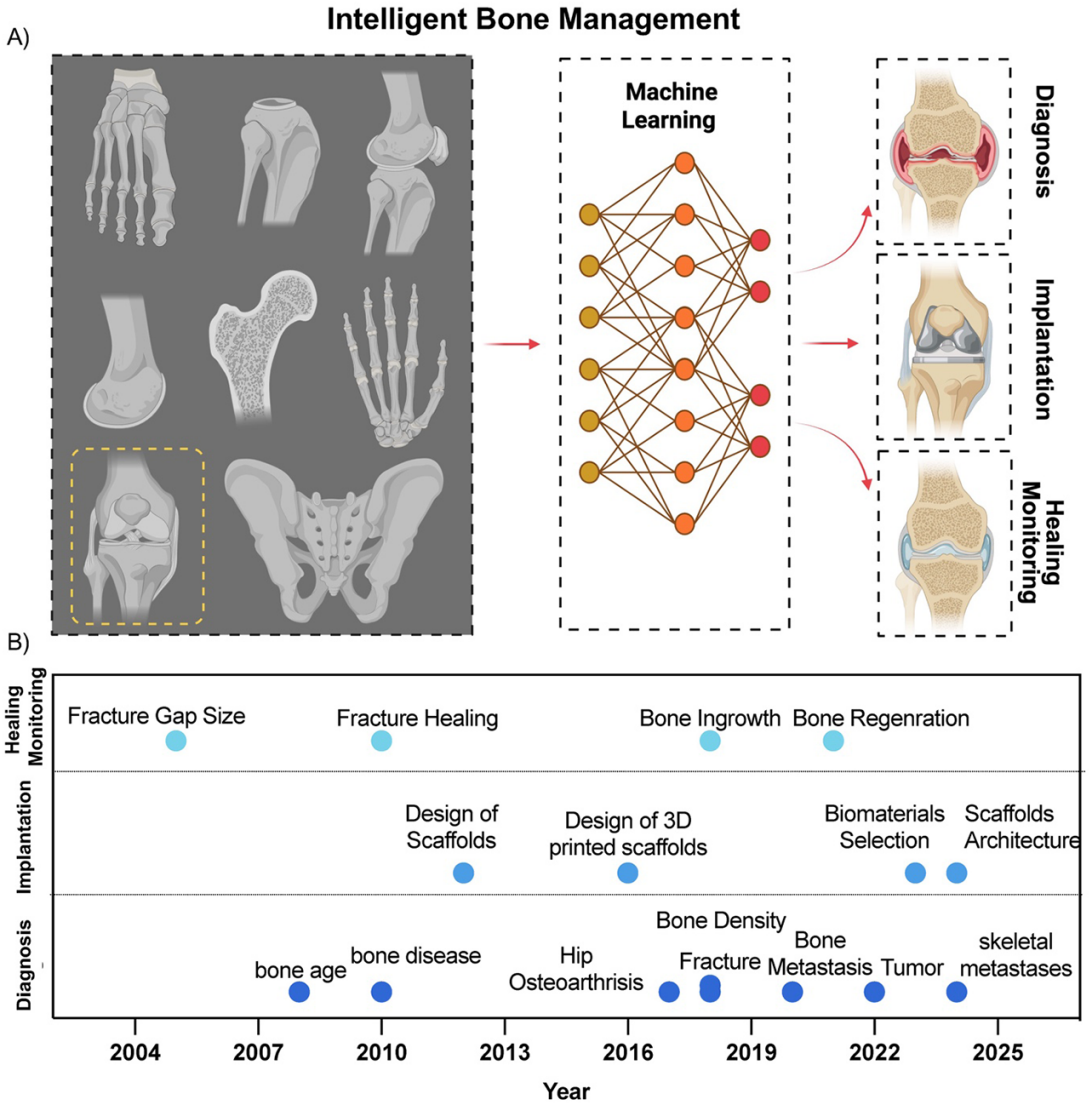
Intelligent orthopedic bone management: workflow and advances. (A) A scheme of ML's assistance in orthopedics for bone management (Created in BioRender. Golafshan, N. (2026) https://BioRender.com/ccnbizj). (B) A timeline of the recent advances in ML's orthopedic applications for bone management: diagnosing bone disease, designing implants, and monitoring the healing process [14, 15, 16, 17, 18, 19, 20, 21, 22, 23, 24, 25, 26, 27, 28, 29].

Recently, the integration of ML into orthopedics has come into the spotlight. Research has emphasized ML's great potential to enhance predictive diagnosis and improve patient outcomes [30]. For example, Park et al. have screened 422 CT datasets to evaluate automated deep learning–based bone mineral density (DL‐BMD) for diagnosing osteoporosis. Their results revealed high diagnostic accuracy for identifying osteoporosis and low bone density through the DL‐BMD method [30]. When it comes to improving the design and selection of biomaterials for implants, Lin et al. have proven ML effective after analyzing the osteoinductivity of biomaterials, developing a substantial database of osteoinductive materials, and using a data enhancement approach to improve model performance [31]. Finally, ML‐driven models are increasingly used to monitor bone healing and its complications. One study showed the potential of an ultrasound imaging deep learning model to monitor bone healing after fracture, which offers simple, rapid, real‐time, and dynamic observation of fracture healing [32].

This paper highlights recent ML advancements and their roles in aiding clinicians in orthopedic treatments. We elaborate on ML roles in improving the intelligent diagnosis of bone disease, optimizing materials and designs for bone regeneration, and managing bone healing and its complications. Furthermore, we will explore the challenges and future perspectives of ML‐driven modern techniques in bone regeneration.

## 2 Bone Defects and Complications

Bone defects are conditions that affect the structural and functional integrity of the skeletal system, often resulting in pain, disability, and a reduced quality of life. Bone disorders arise from different causes, including excessive mechanical forces [33], imbalance between bone formation and bone resorption [34], hormonal imbalances [35], chronic inflammation [36], and infection [37], resulting in gradual or sudden changes in bone structure. In general, bone disorders can be categorized into three different groups: degenerative disease [38], traumatic injuries [39], and bone tumors [40].

Degenerative diseases, including osteoporosis, osteopenia, and osteoarthritis, are characterized by the breakdown of bone and/or joints over time [41]. Specifically, osteopenia and osteoporosis occur due to decreased bone density, caused by an imbalance between bone resorption and formation. Osteoarthrosis is characterized by progressive cartilage degradation and the underlying bone [42]. Traumatic injuries, particularly fractures, stem from a variety of mechanical forces that exceed the structural integrity of the bone. When a force is applied to a bone over a specific area, it creates stress, which in turn causes strain. A fracture occurs when this strain exceeds the bone's elastic limit, leading to deformation and ultimately a break [43]. Another disorder affecting bone tissue is neoplastic disease, which includes tumors. Metastatic and primary bone tumors, such as osteosarcoma, are defined as conditions affecting bone tissue by invading and proliferating at bone sites [44].

Addressing these disorders often requires surgical interventions like inserting implants, which act as structural and mechanical supports [2]. Bone implants (such as autografts and synthetic implants) can possess specific properties that promote bone healing when bone regeneration is not happening on its own [45, 46]. Although current practices are promising, researchers and clinicians are increasingly employing advanced orthopedic methods to train ML algorithms to achieve better outcomes. These practices can contribute to early detection, accurate treatment plans, and real‐time monitoring of the treatment process.

## 3 Machine Learning in Orthopedics

In clinics, human errors adversely affect the precision of diagnoses and predictions. Nowadays, ML can improve decision‐making and accelerate scientific progress in clinics by performing data analysis, making predictions, and enabling automation [47]. To perform these tasks effectively, a clearly defined problem statement is essential. Figure 2 illustrates the main steps in developing an ML model, from identifying the problem statement to the interpretation step [48].

**FIGURE 2:**
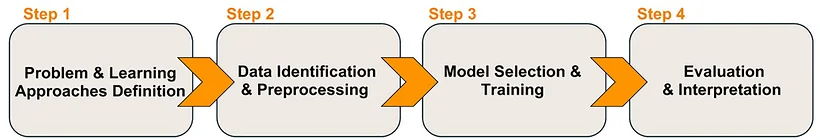
ML model development process: problem identification, data preprocessing, model training, and evaluation.

### 3.1 Definition of Problem and Learning Approaches

The problem statement can be defined based on the model's specific objectives, available data, and its limitations. Afterward, data collection begins, during which the raw measurements or features are fed to the ML model as input data, while the model's prediction or result is considered the output data. For instance, osteoporosis scores, age, gender, and other patient characteristics can be used as input data to predict fracture risk, which is the output in this case. The next step is to select the proper learning approach: supervised, unsupervised, or reinforcement learning.

### 3.2 Data Identification and Preprocessing

Based on the information gathered from patients, the data is categorized into structured, unstructured, and semi‐structured forms. Structured data is information organized in a consistent format, typically in tables like osteoporosis scores. In this data type, preprocessing may involve handling missing values (e.g., osteoporosis scores), standardizing measurements across different sources, and ensuring consistency across coding systems [49]. Unstructured data is information that is not in any specific form or structure, such as MRI and x‐ray images, and its preprocessing step typically involves noise reduction [49]. A combination of structured and unstructured data, semi‐structured data does not follow a strict tabular format, like electronic health records in orthopedics. Here, the preprocessing step harmonizes structured and unstructured data to create a unified dataset [50], resulting in a robust dataset that incorporates all the available information. This dataset follows interrelated, structured algorithms essential for effective model training [51].

### 3.3 Model Selection and Training

Selecting a suitable model depends on the problem type, data quality, and the model's interpretability. ML models can be divided into traditional and deep learnin*g*. Traditional models—including decision trees, support vector machines (SVMs), and linear regression—are effective when the available data is limited and structured, or the relationships between data are relatively simple (e.g., linear or polynomial). However, deep learning models rely on methods that utilize artificial neural networks (ANNs) with multiple layers to model complex patterns [48]. These models are effective when working with large, unstructured, or semi‐structured data, and when the relationships between data are complex, such as nonlinear or spatial. These models include methods like multi‐layer perceptron (MLP), deep neural network (DNN), and convolutional neural network (CNN).

Training the model involves feeding data and helping it learn to recognize patterns with reliable performance. Supervised, unsupervised, or reinforcement learning could be adopted for model training, depending on the selected learning approach. Training supervised learning models can start by randomly dividing the dataset into training, validation (which is optional), and test sets. The model is trained using the input features to learn the mapping between the input and output features. The validation set can be used to evaluate the model during training, making it more reliable. Finally, the model's performance is evaluated using the test set. Unlike supervised learning, an unsupervised learning model uses the entire dataset as input for training, focusing on discovering hidden patterns, structures, or groupings within the training set. However, training a reinforcement learning model focuses on using the whole dataset and interacting with an environment, generating its own data in the form of states, actions, and rewards to maximize the model's cumulative rewards. Simulation is widely used to assess reinforcement learning models, providing a safe, controlled testing and validation framework prior to real‐world applications. Table 1 introduces a summary of ML methods and their applications.

**TABLE 1:** Overview of some ML methods, applications, and limitations.

ML applications	Learning type of ML	Model name	Potential limitations
Predicting bone density in osteoporosis using structured demographic and biochemical data [52].	Supervised	Random Forest (RF), Gradient Boosting (GB)	Explainability and reproducible
Classification of osteoporosis in femoral regions focusing on feature relevance [53]		RUS‐Boosted Trees	Information loss from undersampling
Predicting osteoporosis and Paget's disease using SMILES drug data [54].		Stacked RF, XGBoost	Data diversity, Class imbalance
Prediction of osteoporosis [55]		Logistic Regression, Decision Tree, KNN	Single‐center data
Prediction of osteoporosis risk [56]		CatBoost	No consistently superior algorithm
Predicting infection in patients [57]		Support Vector Machine (SVM)	Limited scalability Sensitive to overlapping data
Optimizing treatment strategies for chronic diseases, such as bone‐related disorders associated with chronic kidney disease [58].	Reinforcement Learning	Quantitative Systems Pharmacology (QSP) model with a Reinforcement Learning approach	Simplified model assumptions, Generalizability, extrapolation
Diagnosing knee osteoarthritis using x‐ray images [59]. Classifying bone tumors in the proximal femur [60].	Supervised	DenseNet, DenseNet201	Dataset (small/possibly imbalanced samples)
Detects fractures (vertebral, cervical) [61] and spinal tumors using X‐rays and MRIs [62].		ResNet, ResNet50	Single‐center dataset
Diagnosing malignant bone tumors around the knee [63].		Vision Transformer (ViT)	Small dataset
Detecting femoral bone tumors in MRI images [64].		Enhanced AlexNet	Explainability
Classifying bone tumors in the tibia and femur using CT images [65, 66].		VGG16‐ViT fusion model	Generalizability (single‐center data)
Segmentation and BMD prediction for osteoporosis in the lumbar spine using LDCT scans [67, 68].		3D U‐Net, DenseNet121	Retrospective study
Classification of femur neck fractures [56]		MobileNetV3	Sensitivity to dataset quality Limited detection of subtle image features
Detection and classification of knee osteoarthritis severity [69]		Faster R‐CNN, AlexNet	Data leakage risk
detection and classification of bone lesions in full‐field radiographs [70]		You Only Look Once (YOLO)	Retrospective design (selection bias risk)
Multimodal MRI analysis for osteoarthritis detection [71].	Unsupervised	Low‐Rank Decomposition Denoising (LRDD)	Small dataset, Overfitting risk

### 3.4 Evaluation and Interpretation

Evaluating the model is essential to assess its generalization performance and ensure the reliability and robustness of the learned patterns beyond the training setting. Specific metrics are used to evaluate the model based on the problem type and input data. Table 2 presents a comprehensive list and description of evaluation metrics. The models’ performance is quantified using metrics such as accuracy, precision, recall, and F1 score. The selection of ML methods is dependent on the input dataset and the prediction outcome.

**TABLE 2:** Common evaluation of metrics across classification, detection, regression, and segmentation [72, 73, 74, 75].

Problem type	Metric	Description
Classification (pre‐defined categories)	Accuracy	Fraction of all correct predictions.
	ROC Curve & AUC	The Receiver Operating Characteristic (ROC) curve shows test accuracy, and the Area Under the Curve (AUC) measures overall performance—closer to 1 indicates better performance.
	F1 Score	F1 Score is the harmonic mean of precision and recall, giving a balanced measure of test accuracy—closer to 1 means better.
	Precision	Percentage of positive results that are truly correct—closer to 1 means fewer false positives.
	Recall (Sensitivity)	Fraction of all actual positive cases that the test correctly identifies—closer to 1 means fewer missed patients.
	Specificity	Proportion of negative cases correctly identified—closer to 1 means fewer false alarms.
	Area Under the Precision‐Recall Curve (AUC‐PR)	Overall measure of the precision–recall relationship—closer to 1 indicates better detection, especially useful for rare conditions.
	DOUBT‐FRACT Threshold (50% sensitivity)	Fixed threshold set to ∼50% sensitivity to flag doubtful fracture cases.
	FRACT Threshold (90% specificity)	Fixed high‑specificity threshold (∼90%) to minimize false‑positive fracture alerts.
	Mean Accuracy	Average of each class's accuracy—closer to 1 means fairer performance across both common and rare conditions.
	Training Loss	Objective value tracked during model training—lower numbers mean the model fits the training data better.
Detection (identifying and localizing specific features or abnormalities)	Average Precision (AP)	Area under the precision–recall curve—higher (closer to 1) indicates better detection, especially for rare conditions.
	Intersection over Union (IoU)	Overlap ratio between the predicted and true areas—higher (closer to 1) indicates more precise localization.
Regression (continuous numerical values)	MAE (Mean Absolute Error)	Average of absolute prediction errors—reports the typical magnitude of deviation in the same units as the measurements.
	RMSE (Root Mean Squared Error)	Square root of the average squared errors—reports the typical error size in the original units, so lower means better fit.
	R^2^ (Coefficient of Determination)	Proportion of total outcome variation explained by the model—closer to 1 means stronger explanatory power.
	MSE (Mean Squared Error)	Mean of the squared differences between predictions and true values—higher values indicate larger errors, with big mistakes penalized more heavily.
Segmentation (delineating specific regions or structures within images at the pixel level)	Dice Similarity Coefficient	Twice the overlap divided by the total segmented area ranges 0–1, where 1 perfectly matches predicted and actual regions.
	Mean IoU	Average of Intersection over Union scores across all classes or images—closer to 1 means consistently strong overlap with true regions.

To interpret ML and deep learning techniques, evaluation metrics are employed. These metrics include accuracy, which typically produces numerical outputs and an overview of performance, but does not clarify how individual predictions are made. Interpretability ensures transparency, reliability, and safe integration in ML models. Deep learning models are considered black box systems due to their complex architectures, making it difficult to interpret how they generate predictions. Interpretation models can be divided into two categories: model‐agnostic and model‐specific, with the former commonly used to interpret black‐box models, including both deep learning and some traditional ML methods. However, model‐specific methods are specifically utilized for deep learning models to interpret complex architectures. These interpretability models can provide insights into feature importance and help users understand which features contribute most to the model's predictions [76]. The following section delves into how ML is employed in orthopedics and how it can help clinicians with bone management.

## 4 ML Advancements in Orthopedics

Some ML models have been used in orthopedic AI‐driven devices to aid clinicians in enhancing diagnostic accuracy [77]. In this section, we will explore the applications of ML in orthopedic clinical practice, with a focus on how it increases the accuracy of disease diagnosis, optimizes material selection and design for bone treatment, and enhances the prediction and monitoring of bone‐healing outcomes.

### 4.1 Intelligent Diagnosis of Bone Disease Using ML

Diagnosing bone diseases using ML‐based models can minimize morbidity and mortality, enhance the quality of life, and help advance medical treatment [78]. Some of the most notable bone diseases that can be aided by ML are degenerative diseases (e.g., osteoporosis and osteoarthritis), traumatic injuries (e.g., fractures), and tumors (e.g., osteosarcoma).

#### 4.1.1 Degenerative Diseases

Considering the subtle and progressive nature of degenerative bone diseases and their nonspecific symptoms, such diseases raise serious challenges. Therefore, early detection, high diagnostic accuracy for proper decision‐making, and patient‐specific treatment planning need to be improved with the integration of ML.

One common degenerative disease affecting millions of people's quality of life is osteoarthritis, but there are no reliable criteria for interpreting its data. To locate and grade knee osteoarthritis on x‐ray images, researchers have developed a deep learning model [69]. This model employed the Faster R‐CNN architecture to pinpoint the knee joint space width (JSW, a measurable metric for osteoarthritis (OA) severity) region in x‐rays, combined ResNet‐50 and transfer learning to extract the features, and utilized a pre‐trained network (AlexNet with transfer learning) to classify the severity of knee osteoarthritis (Kellgren–Lawrence (KL) score). Faster R‐CNN has a great ability to accurately detect and localize small features in complex medical images. By employing ResNet‐50 and transfer learning, this study developed a robust framework for detecting and quantifying JSW.

As shown in Figure 3A, the region of interest detection of minimal knee joint space width was detected in x‐ray images (yellow lines). In Figure 3B, osteoarthritis diagnosis was shown using x‐ray images, with severity classification based on the KL grading system (from normal joint, G0, to severe osteoarthritis, G4). The execution of the proposed work using the learning and training process is depicted in Figure 3C as accuracy versus number of iterations. This suggested model had an automatic classification accuracy of between 99.37% for KL‐G1 and 99.84% for KL‐G0, as shown in Figure 3D [69].

**FIGURE 3:**
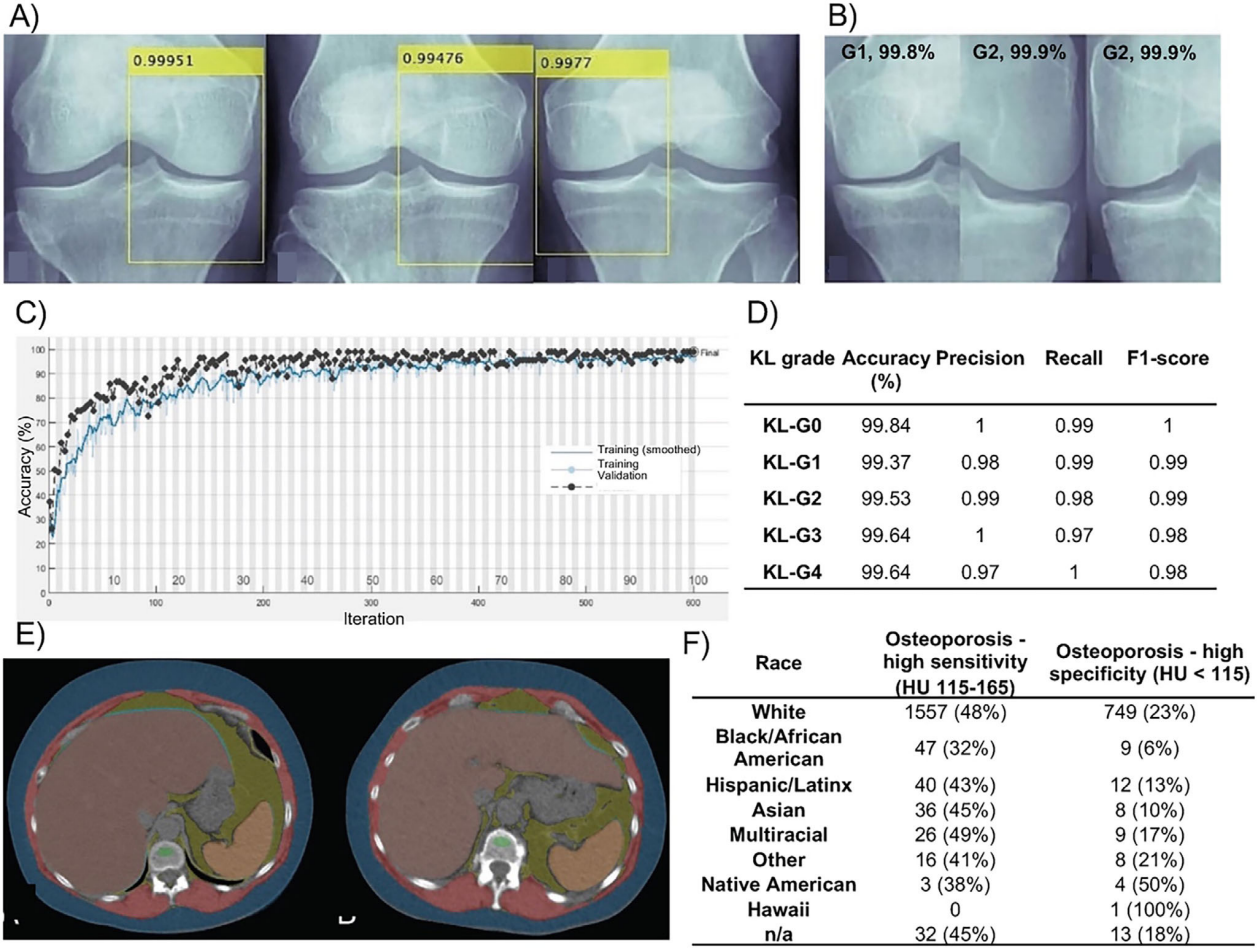
AI applications in diagnosing degenerative bone complications. (A) ROI detection [69]. (B) Diagnosis of osteoarthritis from a normal joint to severe osteoarthritis [69]. (C) Training and validation performance over iterations [69]. (D) AI‐based post‐processing of CT images, Reproduced with permission [69]. 2025, Springer Nature. (E) Left: Axial CT scan at the T12 level segmented by an automated deep learning algorithm for a Hispanic female with osteoporosis (HU 108). Right: Age‐ and BMI‐matched white female with normal trabecular attenuation (HU 212). Trabecular attenuation (green), subcutaneous adipose tissue (blue), visceral adipose tissue (yellow), abdominal and paraspinal musculature (red) [84]. (F) Osteoporosis prevalence across race, Reproduced with permission [84]. 2025, Elsevier.

Similarly, ML methods have been used to predict osteoporosis and osteopenia, two common degenerative diseases in which bone density decreases over time. Researchers have integrated deep learning into an older feature‐based model, thereby developing a fully automated deep learning method to assess bone mineral density (BMD) on 11,035 heterogeneous CT datasets from 11,035 patients. Manual BMD values were obtained from a previous report on the normative L1 trabecular attenuation values. The technical success rate of this new deep‐learning tool (99.3%, or 10 962 of 11 035) was significantly higher than that of the previous feature‐based image processing algorithm (89.4%, or 9,867 of 11 035). This improvement was mainly observed in patients in oblique positions or with conditions such as scoliosis or spinal hardware. The presented Dice score (DS) and average symmetric surface distance (ASSD) for each image indicated that the deep learning tool outdid the feature‐based method in accurately detecting the ROI, particularly in challenging patient positions [79].

An early indicator of bone loss is osteopenia, which was explored in a study using chest CT from 2274 patients to develop the integrated deep learning system. The first phase included 1025 patients to create a model automatically segmenting trabecular vertebral cancellous bone in the T10–T12 vertebrae, using 2 VB‐Net networks. Then, 902 patients were selected to develop and test the classification models. The ternary BMD assessment model was formulated using three deep learning network architectures: DenseNet, ResNet‐18, and ResNet‐50. These models were selected for comparative evaluation to determine the best model suited for CT scans. The Dice similarity coefficient and ROC curve were employed to assess segmentation performance and classify diagnostic performance, respectively. The results of the automatic segmentation model in the first phase showed a mean Dice of over 0.93 in the independent test set. In the second phase, DenseNet and ResNet‐18 performed excellently in detecting bone status, with AUCs of 0.94, 0.91, and 0.98 for DenseNet and 0.96, 0.91, and 0.97 for ResNet‐18 for osteoporosis, osteopenia, and normal BMD, respectively. Nevertheless, the AUC of the ResNet‐50 model for osteopenia was suboptimal (0.76), possibly due to excessive depth and complexity [80]. One potential limitation of ResNet‐50 for osteopenia is that it may involve only subtle reductions in BMD and microarchitectural changes. Also, its reliance on large datasets makes it suboptimal for conditions that require the recognition of localized anomalies, such as osteopenia in CT scans. The selection of the ML model for osteoporosis depends on the imaging modality and the specific purpose. For instance, Random Forest can be beneficial for feature extraction and prediction, while deep learning, such as CNNs, can be useful for image recognition and processing.

A widely used quantitative radiologic technique for assessing BMD and diagnosing osteoporosis and osteopenia is dual‐energy x‐ray absorptiometry (DXA) [81]. Despite being the gold standard for osteoporosis diagnosis, DXA is not available to all people due to its high cost. Thus, in some places, DXA is replaced by Osseus—an inexpensive, noninvasive, portable AI‐driven device using electromagnetic waves. Osseus receives microwave signals crossing the medial phalanx of the patient's middle finger. Combining Osseus results with patient characteristics, including age and sex, helps train ML models, especially Random Forest, to forecast BMD changes and enable early diagnosis of osteoporosis with a sensitivity of 0.853 and a specificity of 0.879 [82].

Although often directly associated with aging, osteoporosis and osteopenia can be related to various diseases, especially metabolic ones. For instance, breast cancer patients have lower BMD and are more likely to develop osteoporosis and osteopenia than healthy people [83]. This has been investigated in a study that considered factors like body composition, age, and the status of hormone receptors when designing a machine‐learning model. Among the developed models, Random Forest demonstrated the best performance, with high accuracy (test set AUC = 0.879) in predicting osteoporosis risk in both breast cancer patients and healthy women [83].

Other than breast cancer patients, ML‐driven predictive models also help assess osteoporosis risk in patients with lung cancer. Research has focused on opportunistic CT scans in patients undergoing lung cancer screening. This study utilized a fully automated ML algorithm to evaluate trabecular bone in 3708 patients, placing ROIs at the T12 vertebrae for attenuation measurements in Hounsfield Units (HU). Figure 3E depicts the annotated single‐slice images of the T12 vertebrae with ROI placement. To detect osteoporosis effectively, a high‐sensitivity threshold (115–165 HU) and a high‐specificity threshold (<115 HU) were used for validation. The results revealed that osteoporosis was more common in females (74% vs. 65% in males, *p* < 0.0001) and whites (72% vs. 49% non‐whites, *p* < 0.0001), as shown in Figure 3F [84]. ML–driven models also assisted in osteoporosis risk assessment in diabetic patients. Unlike conventional prediction tools—including FRAX, QFracture, and Garvan FRC—that have failed to accurately predict fractures because of distinct bone metabolism in such patients, AI models can deliver desirable outcomes [85]. Chen et al. studied 14 419 diabetic patients and integrated XGBoost with a deep neural network (DNN). They demonstrated that the hybrid model combining XGBoost and a Multilayer perceptron (MLP) achieved 90.38% accuracy and 90.52% precision outperforming other ML models [86].

By improving image interpretation and risk prediction, ML has made significant contributions to the early detection of different bone diseases, such as osteoarthritis, osteoporosis, and osteopenia. ML has proved promising in detecting bone involvement in various metabolic disorders that may indirectly affect skeletal integrity. ML‐driven models enhance diagnostic accuracy and patient‐specific evaluations by combining clinical, imaging, and biochemical data. AI‐based tools can also help overcome the restrictions of traditional techniques, especially in subtle and early‐stage cases.

#### 4.1.2 Traumatic Injuries

Besides degenerative bone diseases, ML models can be influential in traumatic conditions, where proper clinical decisions depend on rapid evaluation and outcome prediction.

Diagnosing missed bone fractures, especially in emergency and radiology departments, is a grave concern, but ML can aid physicians in detecting and localizing skeletal fractures on radiographs [87]. In a study, scans of at least 60 examinations per body region—including foot and ankle, knee and leg, hip and pelvis, and hand and wrist—and the validation dataset (*n* = 480) with and without ML assistance were presented to 24 readers. Two musculoskeletal radiologists decided the ground truth, with discrepancies settled by a third. The results showed that ML assistance enhanced fracture detection by 10.4% without lengthening reading time, with sensitivity per patient being 75.2% for AI‐assisted diagnosis and 64.8% for cases without AI aid [87]. ML successfully detected fractures that were overlooked by human readers, demonstrating its superior sensitivity. However, the ML model faced limitations, especially in mislabeling non‐fractures as fractures (false positives) or failing to identify actual fractures (false negatives) [87].

Apart from bone fracture detection, ML algorithms are assisting with more complex diagnostic cases, including the identification of fractures near joint implants, where artifacts and altered anatomy hamper traditional evaluations. For instance, previous research has examined the effectiveness of deep learning models in detecting fractures adjacent to joint implants using a dataset of 1272 x‐rays from peri‐prosthetic femur fractures. The CAM method was used to visualize fracture regions. However, it provides only approximate fracture localization since it focuses on the most discriminant fracture region, as shown in Figure 4A. This research employed four classification models—Densenet161, Resnet50, Inception, and VGG—two object detection models—Faster R‐CNN and RetinaNet—and six confusion matrix‐based measures. This assessment proved that Resnet50 could detect peri‐prosthetic femoral fractures and classify fracture types with accuracies of 95% and 90%, respectively. Although the CAM method provided an approximate visualization of the fracture region, Faster R‐CNN yielded a narrower bounding box for the fracture region (Figure 4B), with a localization accuracy of 78%. Figure 4C compares the Precision–Recall curves of Faster R‐CNN and RetinaNet in identifying peri‐prosthetic femur fractures. Faster R‐CNN shows superior performance, greater accuracy, and fewer false positives, with a precision of 80% and a recall of 98%. In comparison, RetinaNet had lower precision (31%) and recall (97%), indicating more false positives. The higher average precision (AP) for Faster R‐CNN highlights its superior effectiveness in identifying and localizing fractures. The results demonstrated that the implemented approach could be an effective diagnostic tool for fractures adjacent to joint implants [88].

**FIGURE 4:**
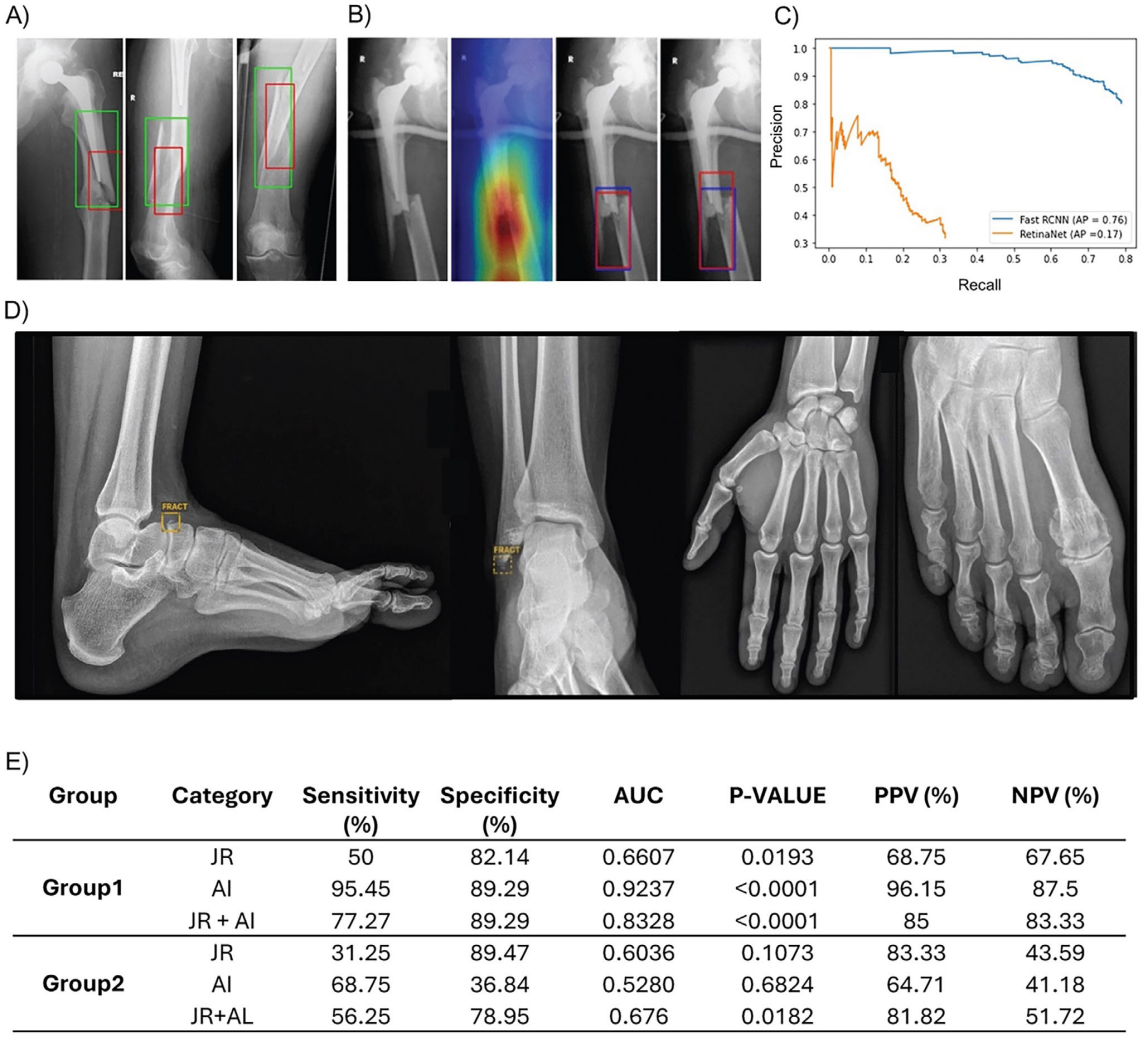
Deep learning applications for fracture detection and localization: qualitative and quantitative evaluation. (A) Illustrates fracture localization using CAM, emphasizing X‐ray areas that the deep learning model prioritizes for identifying fractures. The green box indicates the ground truth, while the red areas represent the model's focus area. Although CAM assists in visualizing fracture localization, the results may not always align well with the ground truth [88]. (B) Instances of fracture classification and localization outcomes (blue: ground truth boxes; red: predicted boxes). These outcomes illustrate the models’ capabilities in fracture localization, with Faster R‐CNN generating more accurate bounding boxes than RetinaNet [88]. (C) Compares the performance of object detection models regarding precision, recall, and localization accuracy, showing Faster R‐CNN outperforms RetinaNet, Reproduced with permission [88]. 2025, Springer Nature. (D) From left to right: First, ML's true response for navicular bone fracture, confirmed by a radiologist. Second, ML's false positive response for external malleolus fracture was confirmed as a calcification by CBCT. Third, ML's true negative response to left‐hand trauma is not a good idea. Fourth, ML's false negative response for lateral sesamoid fracture, confirmed by CBCT [89]. (E) Diagnostic performance of a JR reader with and without AI assistance and AI performance in Group 1 and Group 2, Reproduced with permission [89]. 2025, Ubiquity Press, licensed under CC BY 4.0.

Moreover, ML plays a crucial role in the rapid and accurate evaluation of post‐traumatic bone fractures. Using an ML software package (Boneview Trauma, Gleamer), Dell'Aria et al. detected post‐traumatic bone fractures by investigating the radiographic examinations of 101 patients. Two radiologists (osteoarticular senior: SR; junior: JR) independently assessed the radiographic images, both with and without AI. In this study, AI outperformed the radiologists in diagnosing common features (Group 1), but this was not the case for subtle fractures (Group 2). Figure 4D depicts instances of images processed by the ML software, while Figure 4E displays the positive influence of AI on junior radiologists. In both groups, AI positively affected radiologists’ overall performance, with the increase being significantly greater in the JR group (*p* < 0.05). The findings proved that although reliable for common radiographic fracture detection and useful for radiologists undergoing training, this AI‐driven software cannot outdo osteoarticular senior radiologists, especially in cases of subtle lesions [89].

Additionally, ML can outperform traditional imaging methods when it comes to precisely localizing bone fractures. For example, in one study, an AI system was trained on 60 170 radiographs from patients with bone trauma [90]. The radiologists and physicians were asked to detect and localize fractures with and without R statistical software by highlighting the box around AI‐detected fractures. The results indicated that AI aid increased sensitivity and specificity by 8.7% and 4.1%, respectively, without losing reading speed [90]. Furthermore, Liu et al. developed several ML models to evaluate 8530 patients, including 1090 with osteoporotic fractures. The best performance was delivered by an ML model integrating an SVM and XGBoost. This model exhibited impressive accuracy and precision of over 90% compared with other AI‐assisted models when forecasting osteoporotic fractures. By pinpointing critical risk factors, including blood calcium levels and C‐reactive protein, this model can help clinicians implement early interventions [91].

Finally, with recent advancements in ML, computers can now process and understand complex inputs more accurately and diminish inaccurate diagnoses [92]. Considering the pace at which AI‐driven devices are improving, achieving AI accuracies that significantly surpass those of experienced radiologists is not far‐fetched.

#### 4.1.3 Tumors

ML has great potential in detecting and characterizing bone tumors, including both primary bone cancer and bone metastases. AI‐driven tools can support tumor identification and classification even without prior segmentation. However, segmentation may improve model performance by acting as an attention mechanism, enabling models to focus on clinically relevant tumor regions while reducing background noise. This may explain why segmentation‐based architectures such as Seg‐UNet and Mask2Former outperform those that rely solely on global image features, including Vision Transformer and YOLO‐based models.

ML models can classify x‐ray images to help detect suspicious lesions in patients with bone cancer. For example, researchers have scanned the area around the knee of children with histopathologically verified diagnoses of malignant bone tumors [93]. This study used a pre‐trained Vision Transformer model (with a data split of 60%, 20%, and 20% for training, validation, and testing, respectively) to classify images of healthy and pathological x‐rays [93]. To provide additional insight, Grad‐CAMs were implemented in the final inference step for both healthy and pathological x‐rays, as shown in Figure 5A (top). Based on Figure 5A (bottom), Grad‐CAMs use the gradient information of a deep learning network to reveal the influence of neurons on decision‐making, leading to a colored heat map. By classifying healthy control and pathological groups, the model reached an accuracy, sensitivity, and specificity of 89.1%, 82.2%, and 93.2% in the test groups, respectively. Although this model can detect suspicious x‐rays, thereby decreasing diagnosis time, it cannot identify the potential tumor location [93]. Nonetheless, pairing ML with CT imaging to segment bone tumors automatically is possible.

**FIGURE 5:**
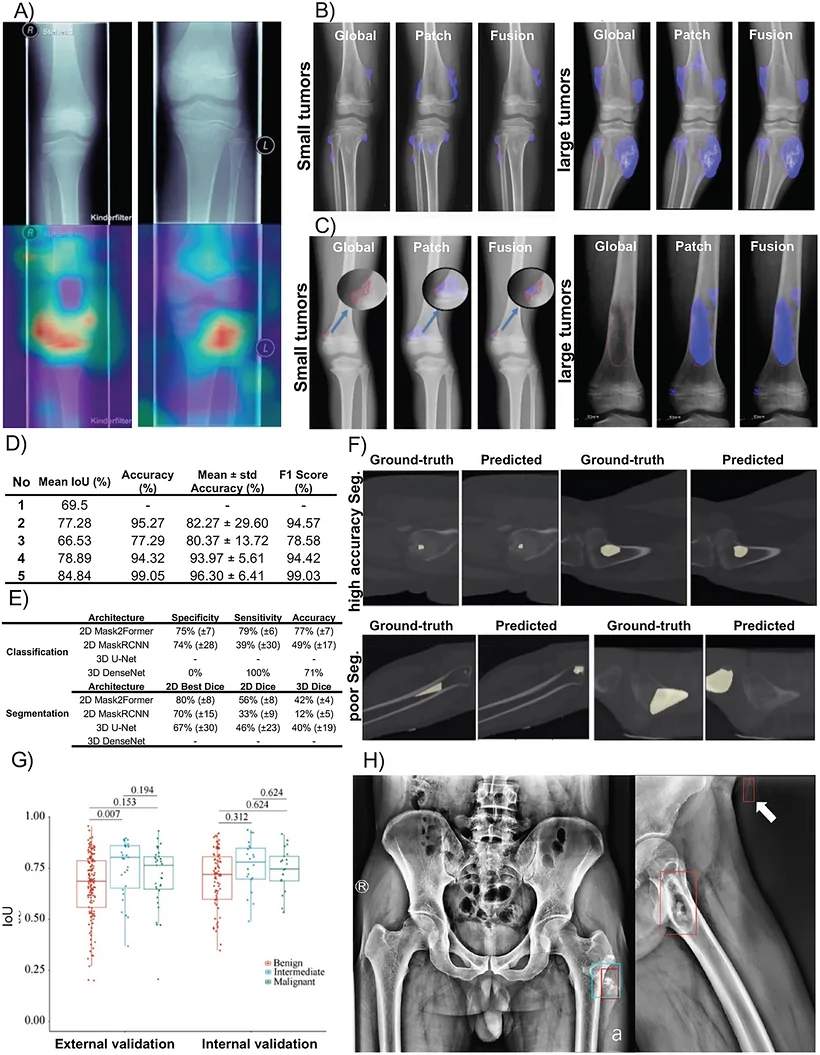
ML in tumor detection. (A) Top row: original x‐rays for a healthy (left) and a cancer patient (right). Bottom row: Grad‐CAMs of the images for healthy (left), displaying more diffuse Grad‐CAMs, and a cancer patient (right), Reproduced with permission [93]. 2025, MDPI AG. (B) Successful cases of the global‐based model improving the fusion outcomes with small tumors (left) and large tumors (right). (red line: ground truth; blue area: tumor detection). (C) Successful cases of the patch‐based model enhancing the fusion results with small tumors (left) and large tumors (right). (red line: ground‐truth; blue area: tumor detection; blue arrow: zoom in the ground‐truth areas). (D) Classification and segmentation performance in the CNUH validation set, Reproduced with permission [94]. 2025, MDPI AG. (E) Bone tumor classification (specificity, sensitivity, accuracy) and segmentation (2D Best Dice, 2D Dice, 3D Dice) in different architectures (2D Mask2Former, 2D MaskRCNN, 3D U‐Net, and 3D DenseNet) [95]. (F) Examples of ground‐truth versus predicted tumor segmentation with high segmentation accuracy even in small tumors (top row) and poor segmentation accuracy (bottom row), featuring masks highlighted in yellow overlaid on sagittal slices, Reproducced with permission [95]. 2025, Springer Nature. (G) Boxplot of IoUs for the three bone lesion subtypes in the internal and external validation set, with the benign and intermediate subtypes having a significant IoU difference in the external validation set but not among the three bone lesion subtypes in the internal validation set [70]. (H) Detection outcomes using YOLO. Left: In an osteochondroma case, the blue box indicates the ground truth, while the red box represents YOLO's predicted ground. The IoU is measured at 0.48. Right: the false‐positive results are demonstrated. The high density of clothes on the radiograph was incorrectly detected as bone lesion by YOLO (the red arrow), Reproduced with permission [70]. 2025, Springer Nature.

Aiming to use ML for bone tumor segmentation, Do et al. have classified and segmented knee bone tumors using a Seg‐Unet as a baseline model to determine normal, benign‐tumor, and malignant‐tumor regions to handle small tumors. Five models were used in this study: Seg‐Unet, Seg‐Unet + ClasSeg, Seg‐Unet + ClasSegDis (Patch‐based), Seg‐Unet + ClasSegDis (Global‐based), and Seg‐Unet + ClasSegDis (Patch + Global Fusion). Figure 5B illustrates the outcomes of successful cases utilizing the fusion results from the global‐based model. Unlike the patch‐based model—which struggled with noise in small tumors, detection of tumors in varying poses, and noise in larger tumors—the global‐based model successfully detected these instances. Figure 5C shows the patch‐based model's successful prediction for both small and large tumors. The integration of both models led to strong performance, as shown in Figure 5D. Model 5, the fusion of the global‐ and patch‐based models, offered the best results (accuracy of 99.05% and mean accuracy of 96.3%) in comparison with Model 2 (with only classification and segmentation branches) and Model 4 (with the addition of a multi‐level distance feature). Additionally, Model 5 presented the best Mean IoU (84.74%) among all models [94].

Unlike previous research, which used a hybrid method with separate global and patch‐based models for segmentation and classification, Potter et al. introduced a unified deep‐learning framework. Using the Mask2Former model, this study combines CT scans with deep learning to automate bone tumor segmentation and classification using 84 femoral CT scans with histologic confirmation of bone lesions [95]. This model exhibited promising results, assisting in clinical decisions, particularly in differentiating between malignant and benign lesions [95]. The model displayed classification accuracy, sensitivity, and specificity of 77% (± 7%), 79%, and 75%, respectively—and a Dice score of 56% (± 8%) evaluating average segmentation performance. As shown in Figure 5E, the Mask2Former model demonstrated improved performance compared with alternative approaches (like MaskRCNN and 3D U‐Net). Also, Figure 5F shows examples of ground‐truth versus predicted tumor segmentation with high and poor segmentation accuracy. This automated system has great potential to facilitate clinical decision‐making (especially optimizing biopsy decisions and minimizing unnecessary procedures) in orthopedic oncology by delivering accurate, efficient, and objective tumor classification [95].

A deep learning model called YOLO was developed in another study to detect and classify bone lesions on radiograph images with minimum manual intervention [70]. This model was trained on 1085 bone tumors and 345 normal bone radiographs to detect bone lesions, classifying them into normal to malignant types in a single, end‐to‐end framework. Based on the detection results, YOLO reached high accuracy (IoU ≥ 0.5), with 86.36% in the internal and 85.37% in the external validation sets. The similarity between the model's IoU scores in both validation sets indicates robust performance and generalizability across datasets. The results show a significantly higher accuracy than radiologists regarding intermediate bone tumor classification (95.73% vs. 88.08%, *p* = 0.004). Moreover, the IoUs were not significantly different between the internal and external validation sets (0.68 ± 0.21 and 0.67 ± 0.17, respectively) or between the three subtypes of bone tumors in the internal validation set (Figure 5G). However, IoUs differed significantly between the benign and intermediate groups in the external validation set, with intermediate lesions exhibiting a higher IoU (Figure 5G). Figure 5H shows the results of YOLO in identifying possible bone lesions. According to IoU metrics, the YOLO‐v5 model effectively detects and localizes bone tumors in both internal and external datasets, with consistent, reliable performance across both datasets. YOLO‐v5 detects and localizes intermediate tumors more successfully than benign tumors, but there were no significant differences between the internal and external validation sets. The findings prove that the YOLO model can aid radiologists in bone lesion detection and classification [70].

Recent deep learning advancements have facilitated the prediction of bone metastasis. A study has utilized a radiomics and deep learning model (DenseNet‐264) to explore bone metastasis in lung cancer patients. To this end, researchers investigated 189 lung cancer patients (100 with and 89 without bone metastasis). Patients with bone metastasis from lung cancer (Figure 6A,B) were used for model training and validation. As shown in Figure 6C, experienced radiologists manually segmented ROIs of primary lung cancer lesions in CT images. Figure 6D shows the performance of radiomics and deep learning models. The DenseNet‐264 model reached an AUC of 0.990 on the training set and 0.971 on the validation set, with these numbers for the radiomics model being 0.815 and 0.778, respectively [96].

**FIGURE 6:**
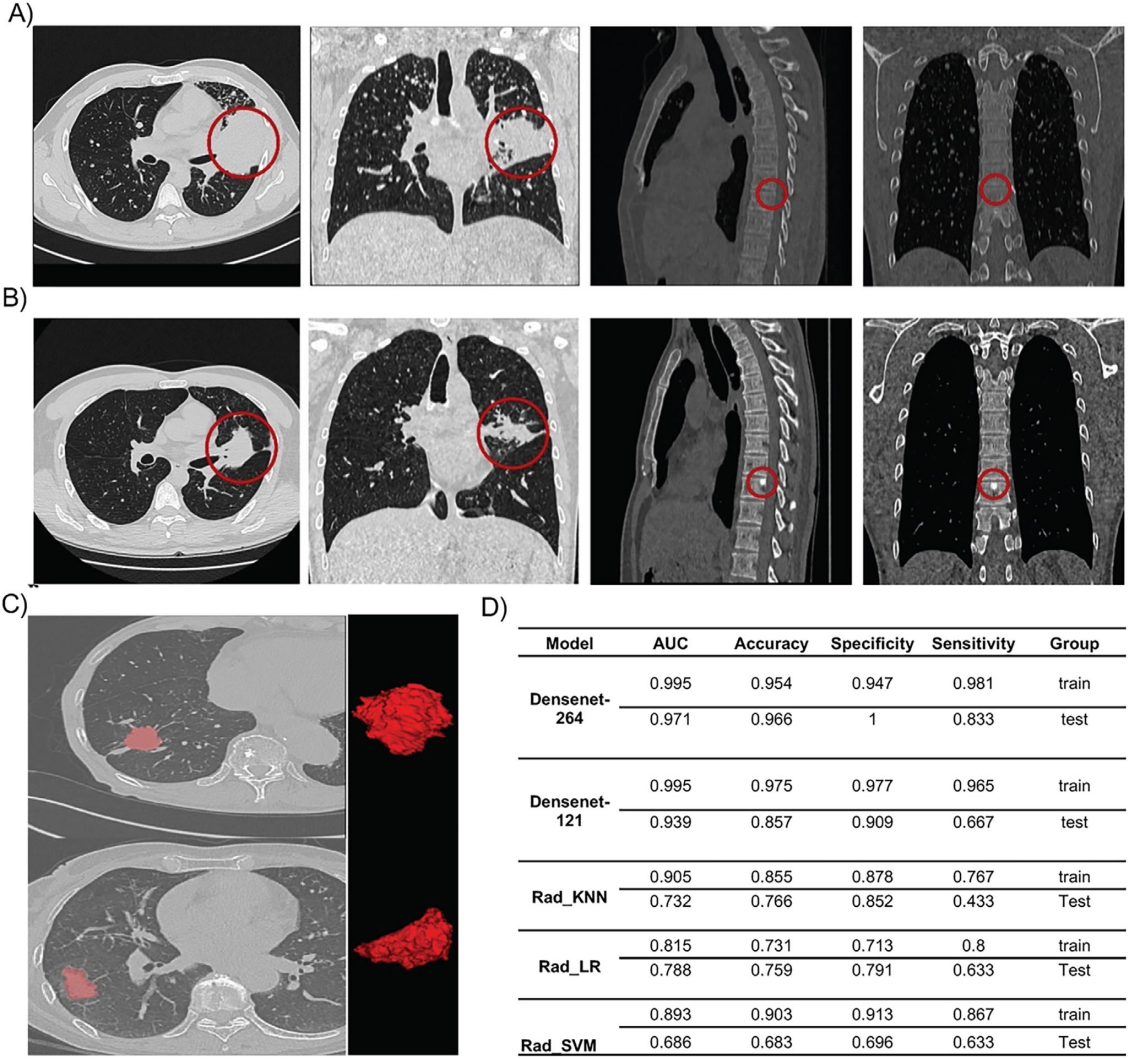
CT scans of a patient with bone metastasis. (A) Initial lung cancer diagnosis. The leftmost image illustrates the lung tumor, while the following images portray the thoracic spine (T10) without evidence of bone metastasis, as indicated by the lack of abnormalities in the red‐circled area [96]. (B) Follow‐up CT scans taken 6 months later. The leftmost image suggests an improvement in the lung tumor post‐treatment. Nevertheless, subsequent scans reveal the development of bone metastases in the thoracic spine (red circles mark the metastatic lesions) [96]. (C) Segmentation of primary lung cancer lesions in chest CT images. A primary lung lesion in a patient without bone metastasis (top) and with bone metastasis (bottom). The left panel displays the CT image with the segmented lesion (red); the right panel shows a 3D reconstruction of the segmented lesion. Skilled radiologists manually delineated the ROIs [96]. (D) Comparison of radiomics and deep learning models, Reproduced with permission [96]. 2025, Elseivier, Licensed under CC BY 4.0.

In summary, as an attention mechanism, segmentation guides the model toward diagnostically relevant areas and improves feature learning. Segmentation is of utmost importance to small, low‐contrast, or complex anatomical parts because it enhances model generalization, classification performance, and clinical interpretability. Therefore, although not mandatory, segmentation is a crucial preprocessing step for analyzing bone tumors with high‐performance, trustworthy AI models.

### 4.2 AI‐Assisted Arthroplasty and Bone Implants

ML applications are not limited to diagnosis; this technology is growing to help optimize bone disease treatment. A major emphasis is on utilizing ML to design and select bone implants. This provides customized, mechanically compatible, and biologically effective solutions. Using ML and computational modeling, researchers can predict the best implant shapes and adjust material properties to improve clinical outcomes. This section examines recent advancements in intelligent bone implants as well as AI's contribution to advancing patient‐specific therapies for bone regeneration.

#### 4.2.1 AI in Arthroplasty Planning

Knee arthroplasty in 60 patients has been studied to compare ML‐based preoperative 3D planning with 2D x‐ray template measurement technology. In the ML group, the CT images were imported into the AI‐KNEE software, which was trained on manually marked feature points and bone markers. The software detects the knee's related physiological and anatomical positions, measures relevant factors, and produces a 3D reconstruction of triaxial alignment. Using a G‐NET neural network, this software detects the femur and tibia, calculates the tibial osteotomy, and predicts tibial prosthesis size. The software spots condylar and Whiteside lines, along with the femoral posterior condylar axis (Figure 7A), in the 3D model. This way, the software predicts the femoral opening point, divides the femoral condyle, and predicts and matches the femoral prosthesis size with the same approach, automatically estimating bone resection volumes. The results of AI‐driven preoperative planning, coupled with postoperative anteroposterior and lateral knee x‐ray simulations, were produced upon completing the final matching. By providing simulated postoperative x‐rays, AI‐Knee enables surgeons to visualize expected implant positioning and alignment prior to surgery. Figure 7B,C depict the AI group's preoperative planning and postoperative review of comparison diagrams. The results proved that the complete coincidence rates for the femoral and tibial prostheses were significantly higher in the AI group (90% and 86.7%, respectively) than in the 2D group (66.7% and 60%, respectively). Figure 7D displays Western Ontario and McMaster Universities Osteoarthritis Index (WOMAC) and American Knee Society (AKS) scores. These scores help evaluate patients’ knee function and recovery in both the 2D template planning group and the AI‐based 3D preoperative planning group at preoperative and 12‐month follow‐up intervals. According to the results, AI‐based planning was more reliable regarding the prosthesis size and axial alignment in total knee replacement [97].

**FIGURE 7:**
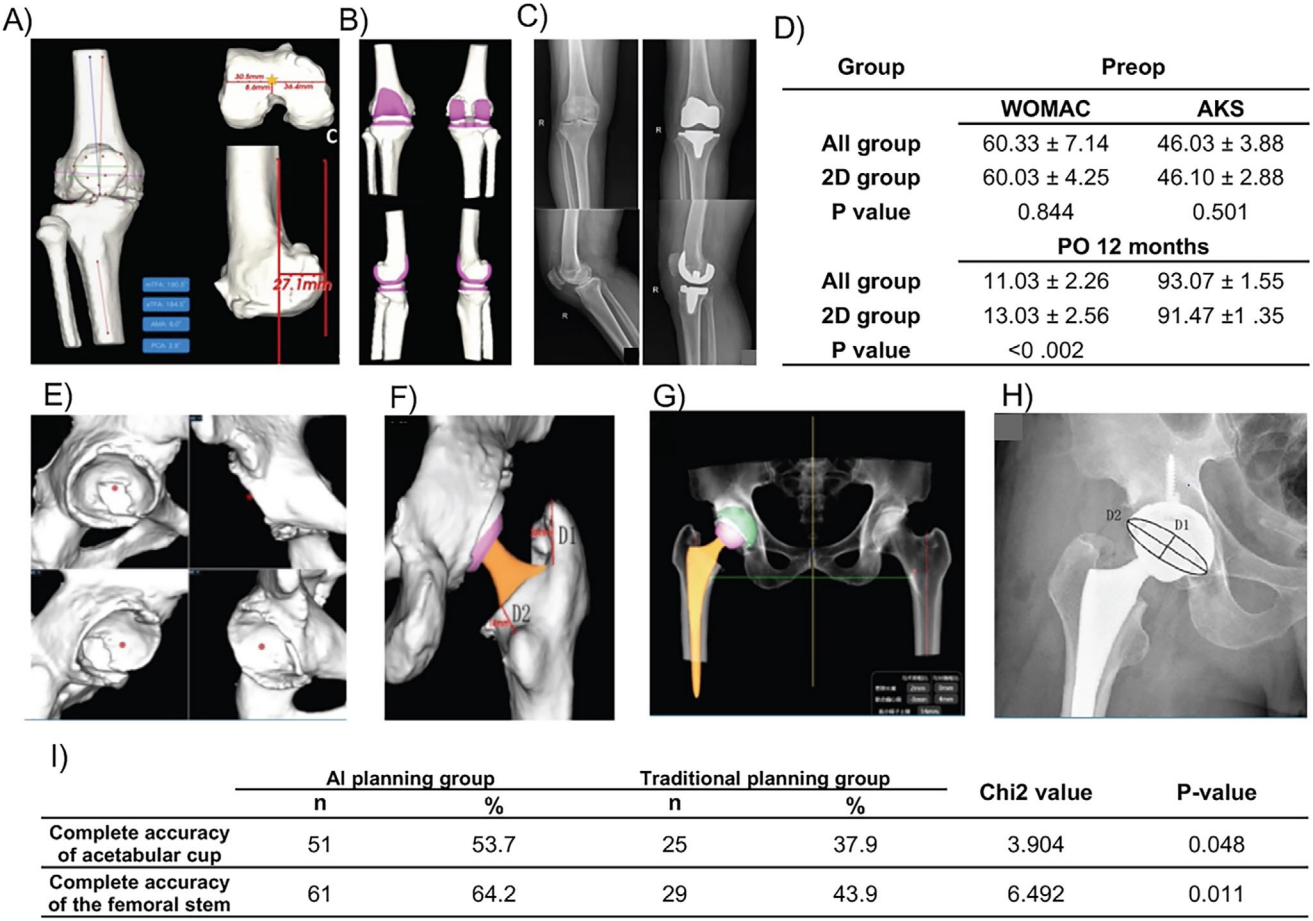
AI‐KNEE: AI‐assisted 3D preoperative planning and postoperative evaluation. (A)–(D). The schematic diagram of AI‐KNEE 3D preoperative planning. (A) Automatic measurement of knee joint parameters and posterior condylar offset [97]. (B) Simulated postoperative x‐ray [97]. (C) Left: preoperative radiograph. Right: radiograph taken 3 days after surgery [97]. (D) Comparison of knee function scores (WOMAC and AKS) at follow‐up, Reproduced with permission [97]. 2025, Springer Nature. (E) AI‐assisted preoperative evaluation [98]. (F) Prosthesis placement and simulation [98]. (G) Postoperative simulation and discrepancy in limb length [98]. (H) Postoperative angle and acetabular positioning [98]. (I) Comparison of preoperative planning accuracy between the AI‐assisted 3D planning group and the conventional 2D x‐ray planning group regarding the acetabular and femoral stem prostheses, Reproduced with permission [98]. 2025, Baycinar Tibbi Yayincilik, licensed under CC BY 4.0.

Lan et al. conducted similar research on total hip arthroplasty to explore the results of AI 3D preoperative planning technology. To this end, they retrospectively assessed 161 patients experiencing total hip arthroplasty (THA), dividing them into the observation group (95 patients treated with AI‐assisted 3D preoperative planning) and the control group (66 patients treated with 2D x‐ray template planning). The AI group used AI‐assisted 3D planning for preoperative design, employing CT images to produce 3D images with AIHIP software. This procedure entailed automatically matching and positioning prostheses using deep‐learning data. Figure 7E–H offer a path from preoperative planning to postoperative simulation, and this emphasizes diminished complication risk, enhanced accuracy, and improved functional recovery. AI‐based technology deepens our understanding of prosthesis sizing, acetabular alignment, and femoral stem positioning. Consequently, AI can make the planning process more efficient while ensuring consistent surgical outcomes. These offer a bright future for orthopedic surgeries. The results presented in Figure 7I revealed that the accuracy of the observation group was significantly higher than that of the control group, with these numbers being 54% and 38% (p = 0.048) for preoperative planning of acetabular prostheses and 64% and 44% (*p* = 0.011) for femoral prostheses. Although the anteversion angle was not significantly different in the observation and control groups (*p* > 0.05), the inclination angle and limb length discrepancy were significantly better in the observation group (*p* < 0.05 for both groups). These show that AI‐assisted 3D preoperative planning outperformed traditional 2D x‐ray template planning [98].

Studies on knee and hip arthroplasty highlight the importance of AI‐assisted preoperative planning. The approach for both knee and hip arthroplasty yields similar results, as AI‐based 3D planning improves implant success compared to 2D planning. These findings support the idea that ML techniques can enable more accurate medical decision‐making and patient‐specific treatment strategies across different joints. However, further studies with multicenter validation are required to confirm the long‐term clinical effectiveness of AI‐based approaches.

#### 4.2.2 AI for Bone Implants and Biomaterials

Studies have also investigated the application of ML to predict the mechanical behavior of 3D‐printed polymer‐based scaffolds to optimize biomaterials for bone tissue engineering. For example, ML was employed in a study to predict the mechanical performance of 3D‐printed polylactic acid (PLA) scaffolds reinforced with calcium hydroxyapatite (cHAP). Among the utilized algorithms, XGBoost and AdaBoost demonstrated high accuracy in forecasting the compressive and tensile strength of the composite, with the XGBoost model's MSE, MAE, and *R* ^2^ scores being 0.0067, 0.0384, and 0.9173 for compressive strength, respectively. These numbers were 0.0087, 0.0594, and 0.8772, respectively, for tensile strength. This reveals the potential of data‐driven approaches for optimizing material properties and developing patient‐specific scaffolds in bone tissue engineering as well as regenerative medicine [99].

Apart from material optimization, ML can assist in identifying the best combinations of biological factors. Using ML‐based techniques, such as ANN, Anastasio et al. uncovered efficient combinations of biological elements, like bone morphogenetic proteins (BMP), to accelerate healing and bone repair. Most orthopedic surgeons utilize only one biomolecular agent (an allograft with embedded BMP‐2). This study, however, evaluated combinations of orthobiologic factors, since they may be more effective than a single agent in a highly multifactorial process like bone fusion. ANN trained with ML on orthobiologic data and their outcomes helped predict the most effective combinations among 2,400 compounds and demonstrated how ML can play a key role in discovering novel biomaterials and developing advanced therapeutic strategies for bone‐related conditions [100].

In another study, Lin et al. dealt with osteoinductive materials used to repair bone defects. They gathered a comprehensive database from the research results in the past 30 years, validated by experts, and used ectopic ossification as a benchmark for assessing biomaterials' osteoinductivity. These researchers adopted a data enhancement approach, reaching an AUC of 0.921, precision of 0.839, and recall of 0.833. Through model interpretation, porosity, BMP‐2, and hydroxyapatite were identified as vital influencers on the results. Having optimized these factors with ML, Lin et al. observed notable enhancements in the rates of new bone formation, with the new bone area ratio in animal experiments (14.7 ± 7%) being significantly higher than the database average (10.97%) [31].

ML can assist researchers in selecting the appropriate materials to bond with bone tissue as well. Among the materials used for bone regeneration, bioactive glass is an effective material that can be used with controlled degradation and ion‐release profiles. For instance, an ML model has been presented to predict the composition‐dependent dissolution behavior of various oxide glasses [101]. Over 1300 data records from glass dissolution experiments were used to train and test the prediction performance of this model. The results showed that this ML model could predict the chemical degradation behavior of the glasses in aqueous solutions over a wide range of pH relevant to their clinical applications [101].

All the above examples clearly highlight how ML is integrated into the development and application of biomaterials for bone disease treatment, paving the way for more tailored and successful treatments. By allowing for precise management of architectural features, mechanical strength, and biological performance, ML technology makes great contributions to scaffold design and material optimization in bone tissue engineering.

### 4.3 Prediction and Monitoring of Bone Healing and Its Complications

The next crucial step is the intelligent monitoring and predictive evaluation of bone‐healing outcomes and associated complications. To this end, ML helps analyze large datasets, providing robust tools for exploring longitudinal clinical and imaging data to assess healing progress. Such models can forecast personalized recovery paths, identify complications early, and lead to adaptive treatment approaches. Incorporating these predictive models into clinical practice can improve patient‐specific care and facilitate making evidence‐based decisions in bone regeneration. This section is divided into imaging‐ and sensing‐based techniques to explore the role of ML in tracking bone repair dynamics and predicting therapeutic outcomes.

#### 4.3.1 Imaging‐Based Predication and Monitoring

For imaging‐based techniques, various modalities (e.g., x‐ray, MRI, ultrasound, and CT scans) can be used to monitor bone healing. The bone‐healing process consists of various stages, for some of which imaging techniques are not suitable. For instance, x‐ray imaging, which is widely used due to accessibility, is limited in detecting early‐stage healing compared to MRI, which provides superior soft‐tissue contrast but is less practical for longitudinal monitoring.

Bulloni et al. explored a ML model using a limited number of evolutional factors obtained from conventional radiological parameters to forecast hip prosthesis failure. They emphasized the evolution of periprosthetic bone and implant positioning over time instead of relying only on the latest radiographic features. To do so, they used 169 annotated radiological features from 162 THA patients, 32 of whom faced implant failure. First, the researchers produced three sets of ML predictors: (i) a standard model using original features, (ii) an evolutional model using derived parameters, and (iii) a hybrid model integrating both, with each set comprising a full and a minimal model (including the most predictive features). Both the evolutional and hybrid models demonstrated high predictive effectiveness (AUC = 0.94) and outperformed the standard model (AUC = 0.84). Interestingly, the minimal model, with only four features (three being evolutional), had an AUC of 0.95, slightly outdoing the full model, which utilized 173 features and had an AUC of 0.94 (Figure 8A). Figure 8B illustrates the subtle limitations of the minimal hybrid model: (i) recurrent dislocation, because this failure type was rare in the training set; (ii) stem loosening, due to the absence of this stem type in the training set; and (iii) metallosis, caused by the absence of clear implant loosening signs. Compared with the full model feature set, the minimal hybrid model demonstrated that three evolutional factors (stem subsidence, resection height reduction, and stem rotation) and a static radiolucency marker capture the biomechanical failure mechanism more efficiently (Figure 8C). The minimal model's high AUC emphasizes the prognostic efficiency of evolutional factors. Figure 8D offers a clinical example visually confirming these quantitative findings. Demonstrating ML models can identify longitudinal radiological changes, especially stem subsidence and radiolucency development, before noticeable implant failure [102].

**FIGURE 8:**
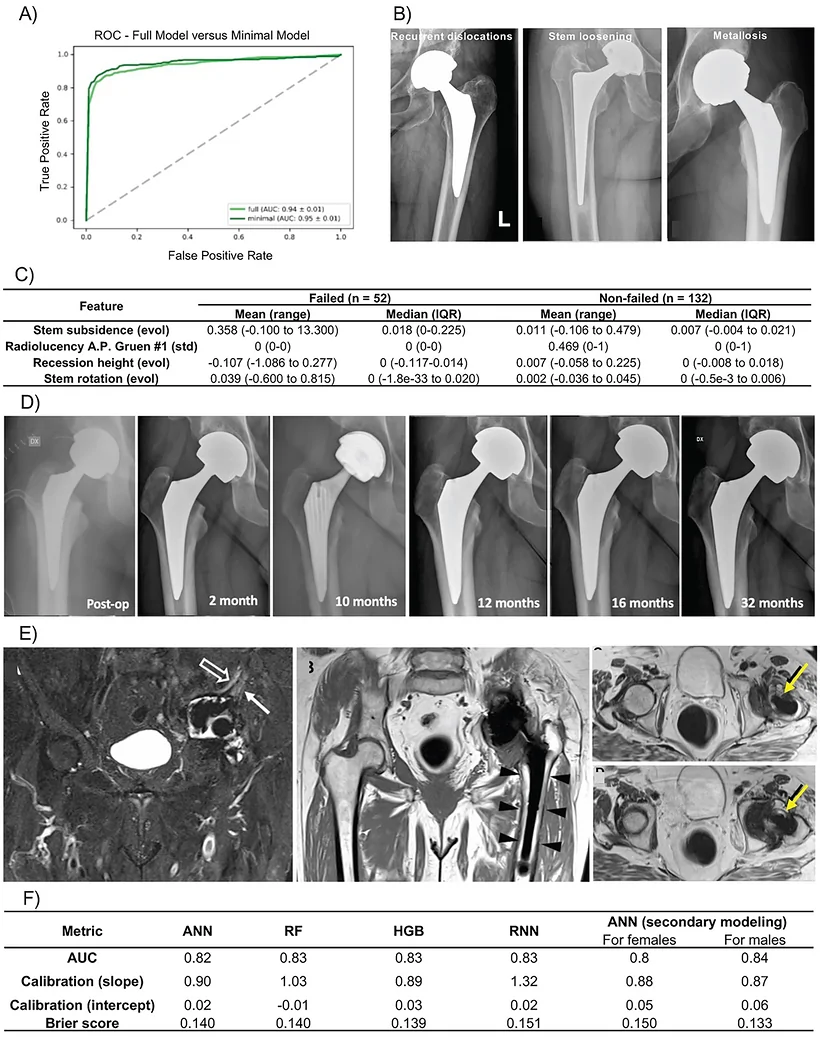
Role of machine learning in THA outcomes: model performance, failure cases, and clinical validation. (A) AUC of the hybrid set in the minimal and full model [102]. (B) Limitations of the minimal hybrid AI model, with misclassified cases such as rare failure modes, unfamiliar implant designs, and non‐radiographic failure mechanisms [102]. (C) Summarized feature values of the minimal hybrid model in the two patient classes [102]. (D) Example of correctly predicted progressive aseptic loosening. In this case, sequential radiographs illustrated increasing stem subsidence, radiolucency, and bone resorption. This confirms the minimal hybrid model's ability to anticipate hip prosthesis failure based on evolutional radiological features, Reproduced with permission [102]. 2025, Springer Nature. (E) Pelvic MRI of a woman with THA infection. From left to right, periprosthetic acetabular bone edema (white arrow) and extracapsular edema (void arrow). Coronal T1‐weighted image shows no bone destruction, and effusion and synovitis are illustrated in axial T2‐weighted images, Reproduced with permission [103]. 2025, Springer Nature. (F) Discrimination and calibration performance of the ML algorithm for the prediction of length of stay during validation. Reproduced with permission [106]. 2025, Springer Nature.

To evaluate fracture healing, it is possible to combine deep learning algorithms with ultrasound imaging. In a study, an ultrasound imaging model was developed using the VGG framework to predict bone healing following fracture, as ultrasound imaging is simple, fast, and nonradiative, and offers real‐time dynamic monitoring of fracture healing. The total number of patients in this research was 164, and data from 104 patients were used to develop the training sets; this number was 30 for each validation and test set. At 2, 4, 8, and 12 weeks after the fracture surgery, the model's AUCs were 0.82, 0.90, 0.86, and 0.67, compared with 0.76, 0.81, 0.81, and 0.86 obtained from manual film reading by radiologists, respectively, with the model's accuracy being 0.73. These findings prove the effectiveness of this monitoring method for bone healing within the 8 weeks following fracture surgery [32]. Here, the drop in AUC after 12 weeks may be due to changes in the fracture‐healing stage, as well as limitations of ultrasound imaging at later stages. At around 12 weeks after surgery, the healing process is usually in the remodeling phase, during which the fracture callus becomes more mineralized and less distinct, making it more difficult to detect clear structural differences with ultrasound. In addition, ultrasound images may be less sensitive to subtle changes in bone structure at this stage than earlier healing periods. These factors together may explain the decrease in model performance at 12 weeks. To improve accuracy during bone remodeling, x‐ray imaging can be used to achieve higher ML performance during healing. Using comprehensive datasets and pattern recognition, such AI‐driven systems can improve diagnostic accuracy and provide clinicians with quantitative insights into bone repair progression.

Machine learning also plays a crucial role in predicting post‐surgical infections after bone surgery. For example, research has used preoperative MRI images to determine whether ML can distinguish septic from nonseptic THA failure. The study comprised 173 patients undergoing first‐time THA revision surgery after preoperative pelvic MRI, of whom 117 were used for the internal training/validation testing cohort and 56 for the external testing cohort. An SVM algorithm was designed using MRI images to predict THA infection, integrating a nested fivefold validation method. The external cohort was utilized to assess the ML model's performance. In the internal training cohort, the SVM classifier attained 92% sensitivity, 62% specificity, 79% positive predictive value, 83% negative predictive value, 82% accuracy, and 81% AUC in forecasting THA infection. Key predictors were bone edema, extracapsular edema, and synovitis. A representative case of this study is shown in Figure 8E. In the external testing cohort, the SVM classifier maintained 92% sensitivity, 79% specificity, 89% positive predictive value, 83% negative predictive value, 88% accuracy, and 89% AUC. These findings demonstrate that an SVM‐based ML model has the potential to aid radiologists in predicting THA infections based on MRI features [103].

#### 4.3.2 Sensing‐Based Prediction and Monitoring

Sensing‐based techniques collect data and monitor data across various bone‐healing stages. Although studies have used the dataset to predict bone healing, the performance of ML models varies across the healing process. This remains a challenge due to subtle physiological changes and limited detectable features in conventional imaging modalities. Therefore, obtaining a biologically relevant dataset in a time‐series manner would be beneficial for overcoming this challenge.

Liu et al. developed a three‐dimensional computational model to assess healing stages in distal radius fractures by combining mechano‐regulated cell differentiation, tissue formation, and angiogenesis. Having been validated with clinical data, this model generated 3600 clinical data points to train the ML algorithms, which helped identify optimal algorithms for different healing stages. According to the results, the optimal algorithm varies with the healing stage, with the cubic SVM and trilayered ANN having the best predictive performance during the early and late healing stages, respectively. Findings suggest that while Smith fractures with medium gap sizes can enhance healing by promoting larger cartilaginous callus formation, Colles fractures with larger gap sizes may be associated with delayed healing due to excess fibrous tissue development. This research highlights the importance of choosing appropriate ML algorithms based on the healing stage before implementation in clinical applications [104].

Another study has concentrated on bone non‐union, one of the serious complications in trauma surgery, influencing 10%–30% of fracture cases. Since treating these non‐unions is complicated and may result in amputation, an accurate prognosis is critical for patient care, which can be enabled using ML, even when datasets are limited. This study employed a dataset of 797 patients with long bone non‐union and three ML models—logistic regression, SVM, and XGBoost. The models achieved 70% sensitivity and specificities of 66%, 49%, and 43% for XGBoost, SVM, and logistic regression, respectively. According to the results, it is feasible to diagnose patients at risk of unsuccessful healing after initial surgical revision treatment at early stages [105]. Thus, ML models have the potential to aid in the early identification of severe bone‐related complications.

Machine learning applications are not limited to predicting post‐surgical infections, but extend further to predicting prolonged hospital stays, which are worth consideration given the high healthcare costs and readmission rates. Chen et al. have evaluated the effectiveness of ML algorithms in the prediction of prolonged length of stay. To this end, they assessed 11 737 cases after revision THA using four ML models, namely ANN, RF, histogram‐based gradient boosting (HGB), and KNN. Key predictors in this research comprised preoperative blood tests (hematocrit, platelet count, leukocyte count), preoperative transfusion, operation time, reasons for revision (infection), and patient age. All models demonstrated clinical utility. However, according to Figure 8F, ANN attained the best predictive accuracy and calibration for prolonged length of stay. The developed models can help clinicians stratify patients based on key factors, enhance care coordination, and optimize discharge planning and costs [106].

It should be noted that imaging modalities provide robust structural assessment of bone healing, particularly in later stages. In comparison, sensing‐based techniques offer earlier insights into underlying physiological processes but are subject to greater variability and limited standardization. Integrating imaging‐ and sensing‐based techniques through multimodal ML approaches may enhance predictive performance in bone healing.

In summary, this section discussed three primary scopes in which ML can assist patients: diagnosis of bone disease, optimization of the design and materials for bone implants, and prediction and monitoring of bone healing and its complications. If used properly, ML has the potential not only to help restore bone structure and function but also to offer personalized medical treatment, thus decreasing mortality and improving patients’ quality of life.

## 5 Challenges and Future Perspectives

The application of ML in healthcare is increasing rapidly. However, integrating ML into orthopedics raises several concerns. Some of the ongoing clinical and ML‐related challenges fall into three categories: (i) data‐related concerns, (ii) clinical translation barriers, and (iii) regulatory considerations.

### 5.1 Data‐Related Concerns

Despite the availability of structured and unstructured data sources, several challenges impact the performance and generalization of ML models in clinics. These challenges can be categorized into class imbalance; cross‐institutional domain shifts; heterogeneity across datasets; and limited availability of large, well‐annotated datasets.

Class imbalance remains a common challenge in orthopedic datasets, potentially limiting their ability to encompass the full range of patient populations. To put it simply, when one class greatly outnumbers another, the ML model learns the majority class better. This reduces not only sensitivity and recall for minority classes but also the detection of rare conditions (such as bone tumors and implant failures). Class imbalance also affects generalizability, as the ML model learned a biased class distribution. Thus, performance may decline when the model is applied to a patient population with a different disease prevalence than the population on which it was trained. This is why most AI models overfit to demographics and misclassify rare but clinically important underrepresented groups, limiting the personalization of treatments [107]. To handle this problem, a wider variety of patient data [108] and self‐supervised learning techniques [109] can be used to improve model robustness and generalization [108]. Selective prediction techniques and the integration of multi‐omics data with healthcare records are other ways to administer patient‐specific treatments [108].

As for data availability, cross‐institutional domain shifts pose a challenge for clinics. Data collected across different hospitals often varies due to differences in patient demographics, device variability, data format protocols, and data imbalance across institutions, which affects the performance of ML models. These inconsistencies in reporting result in varying healing outcomes in orthopedic settings [110]. In fact, a model trained at one institution may show a drop in accuracy or AUC when used elsewhere, decreasing its clinical generalizability. Addressing these shifts requires strategies such as domain adaptation techniques, harmonized data collection protocols, and multicenter collaborative training to ensure that ML models perform reliably across diverse clinical settings.

Another challenge in ML modeling is heterogeneity across datasets. AI models should consider diverse datasets (such as imaging and clinical records), numerous biological interactions, complex cellular communication, and individual variability in biological responses to treatment [100]. These orthopedic datasets differ in imaging modality (MRI, CT, x‐ray), scanner type, image quality, disease severity, and patient demographics, which not only adversely affect performance and generalizability but also complicate the modeling process. To elaborate, greater variability makes pattern recognition more challenging and decreases the consistency of model prediction. It also prevents features learned from one dataset from being successfully transferred to another dataset with different characteristics. Nevertheless, progress in AI, especially data normalization and multimodal learning, will facilitate the integration of different data types [111]. Using imaging technologies, AI‐driven models have the potential to predict disease progression and bone healing for each patient [112]. Solving the challenges raised by biological complexity also calls for multidisciplinary collaboration among biologists, chemists, materials scientists, and computer scientists [113]. Although beneficial, these collaborations often encounter obstacles due to data heterogeneity because combining data from different sources with varying formats and quality standards can be technically complex [114]. The absence of similar terminology and methodologies across fields can create communication barriers, which necessitates effective coordination and translation efforts, as well as updated knowledge of AI tools for healthcare professionals.

We should also consider the limitations of obtaining large, annotated, and standardized datasets for training and reforming ML algorithms [115]. Additionally, annotation of medical images requires radiologists and orthopedists, making it difficult to collect large datasets. Lack of access to large, well‐annotated datasets increases the risk of overfitting and prevents the model from learning meaningful patterns. This means that, despite performing well on the training dataset, the ML model may perform poorly on unseen data, which hinders AI's ability to deliver accurate diagnostics and treatment suggestions [116]. To develop powerful, accurate, and generalizable AI models, data sharing and collaboration across institutions are critical [117]. In this regard, cross‐training CNNs across various datasets can help gather data from different institutions [118]. These, along with standardized reporting practices [119] and data augmentation methods, such as geometric transformations and latent diffusion models [120], can also be adopted to ensure large datasets, diversity, and comprehensiveness [118]. Another promising solution is generative AI models, including GANs and VAEs, which produce synthetic datasets that augment existing data to develop AI models [121]. For example, synthetic x‐ray images obtained from CT scans have improved the robustness of AI models in bone segmentation tasks [122]. Despite these advancements, strict regulations for protecting patient information [123, 124] limit access to the necessary data for training AI models [125]. The privacy issue can be solved by developing privacy‐preserving methods [123, 124] like federated learning, which allows ML models to be trained without transferring data [126], and differential privacy, which involves adding noise to parameters to hamper the identification of individuals in datasets.

### 5.2 Clinical Translation Barriers

There are several practical barriers to the clinical translation of ML approaches. Some of these barriers include integration into existing clinical workflows, building trust among healthcare professionals, and addressing algorithmic biases [127]. Addressing these concerns requires clear standards for AI research, robust validation, and transparency in model design.

Integrating the ML system into the clinical workflow has significant challenges. ML systems should integrate with existing radiology and orthopedic clinical tools (e.g., electronic health records). However, due to AI models’ “black box” nature, AI‐driven recommendations lack explainability and transparency; that is, the reasoning behind model decisions is often opaque and difficult to interpret. Since clinicians and patients cannot fully understand how decisions are made, their trust in and acceptance of AI erode, reducing the likelihood of adoption in real‐world practice [128, 129]. To maximize the benefits of ML models and encourage their adoption, seamless integration and trust‐building between healthcare professionals are necessary.

Seamless integration of AI models and trust‐building can be achieved if AI experts and clinical professionals employ explainable AI (XAI). This is because these models increase transparency in decision‐making processes, making AI systems more interpretable and enabling healthcare professionals to verify whether AI‐driven recommendations are validated [130, 131]. This interpretability improves clear communication and human‐AI synergy while offering excellent diagnostic performance [131, 132].

Another challenge is algorithmic bias. ML modeling requires large volumes of clinical data, but the limited size of the training dataset may not adequately represent all patient populations, disease severities, or clinical institutions. As a result, model performance may vary across different patient groups, limiting generalizability. To overcome this challenge, it is necessary to encourage multicenter collaborations, standardized data collection protocols, and secure data‐sharing frameworks.

### 5.3 Regulatory Considerations

Before implementing AI systems in clinical practice, regulatory approval is essential to validate and demonstrate their safety and effectiveness [133]. Nonetheless, traditional approval frameworks are designed for static models. This means that these frameworks may not suitably account for continuous‐learning systems whose performance can evolve after deployment. The regulatory gap created by these continuous‐learning models shows the need for clear regulatory frameworks and ongoing monitoring. AI regulatory frameworks require careful consideration of ethical, technical, societal, and practical factors. However, this results in slow, complicated, unpredictable, and inconsistent regulations that lag technological breakthroughs [134, 135].

Although recent initiatives such as EU AI Act indicate progress in AI applications in healthcare [136], a comprehensive regulatory framework is still needed. Such a framework should establish clear requirements for validation, transparency, accountability, and post‐deployment monitoring while maintaining high standards for safety and efficacy [137, 138]. Consequently, collaboration among policymakers, regulatory agencies, healthcare professionals, and AI developers is essential to ensure the responsible and safe integration of AI technologies into clinical practice.

## 6 Conclusion

This review paper explored the role of ML in three major areas of orthopedic bone care: (i) diagnosis of degenerative diseases, traumatic injuries, and bone tumors; (ii) optimization of intelligent bone implant designs and materials; and (iii) prediction and monitoring of bone healing and its common complications. Considering these applications, ML is not a mere experimental tool. Instead, it can engage with all clinical processes, ranging from initial detection to long‐term follow‐up. This paper indicated that if ML models are trained on comprehensive, accurate data, they can make significant contributions to orthopedic bone care. For instance, ML models can enhance the reliability and sensitivity of diagnostic evaluations; aid in creating implants that meet each patient's requirements; and identify patients at high risk for delayed healing, nonunion, or other complications. Besides assisting in restoring bone structure and function, the thoughtful integration of ML into current workflows could promote patient‐specific treatments and closer, data‐driven monitoring of the recovery process. Nevertheless, the existing evidence is still inconsistent. This is because many ML models rely on small, limited datasets; lack external validation; and are primarily tested in retrospective settings. To achieve genuine clinical impact, we should consider different factors—including rigorous validation; transparency in reporting; consideration of bias and hasty generalizations; and strong collaboration among clinicians, scientists, and engineers. In summary, ML should not be considered a substitute in orthopedics, but rather a supportive technology. Effective use of ML can enhance clinical diagnostic decision‐making, promote more intelligent implant designs, and enhance the prediction and monitoring of bone healing and its common complications. All of these can ultimately lead to reduced complications and mortality rates while improving the patients’ quality of life.

## Conflicts of Interest

The authors declare no conflicts of interest.

## Data Availability

Data sharing not applicable to this article as no datasets were generated or analysed during the current study.
